# Supplementary material for: Genetic models reveal historical patterns of sea lamprey population fluctuations within Lake Champlain
Source: PeerJ. 2015 Oct 29;3:e1369. doi: 10.7717/peerj.1369 (PMC4631463; doi:10.7717/peerj.1369)
Supplement: Table S1 — Five chains were run for this set of parameters (see main text for more detail). With the exception of mutation rate, broad priors were used for the parameters to test whether the model could detect population changes from the data. [file peerj-03-1369-s001.docx]

**Table S1.** Input for prior distributions in the hierarchical Bayesian coalescent model MSVAR v1.3 (Storz & Beaumont, 2002). Five chains were run for this set of parameters (see main text for more detail). With the exception of mutation rate, broad priors were used for the parameters to test whether the model could detect population changes from the data.

| **Parameters** | | **Input** |
| --- | --- | --- |
| Historical Size (N1) | | |
|  | M^1^ | 5.08 |
|  | V^1^ | 1 |
|  | α^2^ | 5.08 |
|  | σ^2^ | 3 |
|  | β^3^ | 0 |
|  | τ^3^ | 0.5 |
| Current Size (N0) | | |
|  | M^1^ | 5.08 |
|  | V^1^ | 1 |
|  | α^2^ | 5.08 |
|  | σ^2^ | 3 |
|  | β | 0 |
|  | τ | 0.5 |
| Mutation Rate (μ) | | |
|  | M^1^ | -3.3 |
|  | V^1^ | 0.5 |
|  | α^2^ | -3.3 |
|  | σ^2^ | 0.5 |
|  | β^3^ | 0 |
|  | τ^3^ | 2 |
| Time Interval (t) | | |
|  | M^1^ | 4.04 |
|  | V^1^ | 1 |
|  | α^2^ | 4.04 |
|  | σ^2^ | 3 |
|  | β^3^ | 0 |
|  | τ^3^ | 0.5 |

^1^ Priors for the mean (*M*) and variance among loci (*V*) of the corresponding parameters reported as log10-transformed values.

^2^ Hyperpriors used to model the prior log_10_ distribution for *M* with the mean (*α*) and variance (*σ*) drawn from a normal distribution*; α* and *σ* are reported as log_10_-transformed values.

^3^ Hyperpriors used to model the prior log_10_ distribution for *V* with the mean (*β*) and variance (*τ*) drawn from a normal distribution truncated at zero.
