# Supplementary material for: Genetic models reveal historical patterns of sea lamprey population fluctuations within Lake Champlain
Source: PeerJ. 2015 Oct 29;3:e1369. doi: 10.7717/peerj.1369 (PMC4631463; doi:10.7717/peerj.1369)
Supplement: Table S2 — Three replicates of each altered prior were run in BEAST and analyzed using Tracer. Manually altered priors are shaded. Replicates yielded similarly patterned BSPs. Effective population size in 2009 and the range of time when the credibility interval was within 95% HPD were used to compare each run. [file peerj-03-1369-s002.docx]

**Table S2**. Bayesian Skyline Plot Sensitivity Analysis of Lake Champlain samples. Three replicates of each altered prior were run in BEAST and analyzed using Tracer. Manually altered priors are shaded. Replicates yielded similarly patterned BSPs. Effective population size in 2009 and the range of time when the credibility interval was within 95% HPD were used to compare each run.

| **Parameter** | **Baseline** | **Population Altered 1** | **Population Altered 2** | **Rate Altered** |
| --- | --- | --- | --- | --- |
| Max pop size | 10,000 | 20,000 | 100,000 | 10,000 |
| Clock rate | 3.6x10^-8^ | 3.6x10^-8^ | 3.6x10^-8^ | 2.0x10^-8^ |
| N_E 2009_ (stdev) | 3,145 (±1,199) | 6,624 (±24) | 32,618 (±1,199) | 3,180 (±135) |
| 95%HPD (stdev) | 156 (±88) | 122 (±1) | 420 (±173) | 103 (±45) |
